# How New Subscribers Use Cancer-Related Online Mailing Lists

**DOI:** 10.2196/jmir.7.3.e32

**Published:** 2005-07-01

**Authors:** Barbara K Rimer, Elizabeth J Lyons, Kurt M Ribisl, J Michael Bowling, Carol E Golin, Michael J Forlenza, Andrea Meier

**Affiliations:** ^3^School of Social WorkThe University of North CarolinaChapel Hill, NCUSA; ^2^Lineberger Comprehensive Cancer CenterThe University of North CarolinaChapel Hill, NCUSA; ^1^Department of Health Behavior and Health EducationSchool of Public HealthThe University of North CarolinaNCUSA

**Keywords:** Internet, cancer, patients, survivors, online communities, mailing lists, online support groups, listservs

## Abstract

**Background:**

Online cancer-related support is an under-studied resource that may serve an important function in the information seeking, care, and support of cancer patients and their families. With over 9.8 million cancer survivors (defined as anyone living with cancer) in the United States alone and the number growing worldwide, it is important to understand how they seek and use online resources to obtain the information they need, when they need it, and in a form and manner appropriate to them. These are stated cancer communication goals of the US National Cancer Institute.

**Objectives:**

Our purposes are to (1) present background information about online mailing lists and electronic support groups, (2) describe the rationale and methodology for the Health eCommunities (HeC) study, and (3) present preliminary baseline data on new subscribers to cancer-related mailing lists. In particular, we describe subscribers' use of mailing lists, their reasons for using them, and their reactions to participating shortly after joining the lists.

**Methods:**

From April to August 2004, we invited all new subscribers to 10 Association of Cancer Online Resources mailing lists to complete Web-based surveys. We analyzed baseline data from the respondents to examine their perceptions about cancer-related mailing lists and to describe how cancer patients and survivors used these lists in the period shortly after joining them.

**Results:**

Cumulative email invitations were sent to 1368 new mailing list subscribers; 293 Web surveys were completed within the allotted time frame (21.4% response rate). Most respondents were over age 50 (n = 203, 72%), white (n = 286, 98%), college graduates (n = 161, 55%), and had health insurance (n = 283, 97%). About 41% (n = 116) of new subscribers reported spending 1 to 3 hours per day reading and responding to list messages. They used the mailing lists for several reasons. Among the most frequently reported, 62% (n = 179) strongly agreed they used mailing lists to obtain information on how to deal with cancer, 42% (n = 121) strongly agreed they used mailing lists for support, and 37% (n = 109) strongly agreed that they were on the mailing lists to help others. Smaller proportions of new subscribers strongly agreed that others on the mailing lists had similar cancer experiences (n = 23, 9%), that they could relate to the experiences of others on the lists (n = 66, 27%), and that others on the list gave them good ideas about how to cope with cancer (n = 66, 27%).

**Conclusions:**

Cancer-related online mailing lists appear to be an important resource, especially for information seeking but also for support of cancer survivors. These were the primary motivators most members reported for joining mailing lists. The modest proportion of subscribers who strongly agreed that they could relate to others' cancer experiences (as well as similar responses to other process questions) is undoubtedly due at least in part to the short duration that these subscribers were involved with the mailing lists. Emerging data, including our own, suggest that mailing lists are perhaps under-used by minority patients/survivors. These preliminary data add to a growing body of research on health-related online communities, of which online mailing lists are one variant.

## Introduction

### Use of the Internet for Health Information

Recent data indicate that 65% of men and 61% of women in the United States go online [1]; the average American spends over 11 hours online each week [2]. Moreover, it is estimated that 56.3 million people in the United States actively seek online information about chronic diseases [3], and 74% of all US adults who use the Internet report that they had looked for health information online in 2004 [4]. By 2005, it is estimated that approximately 88.5 million adults will use the Internet to seek online health information (eHealth) [5]. Although some researchers have questioned the precision of these estimates, it is clear that millions of people use the Internet for health information and that the Internet is an increasingly important health information source [6-8].

By 2001, there were approximately 9.8 million cancer survivors in the United States [9]. Since survivors are defined as anyone living with or surviving cancer, this is a large population of potential eHealth users. Understanding how they seek and use online resources is important if we are to assure that they have the information they need, when they need it, and in a form and manner appropriate to their needs. These are communication goals of the National Cancer Institute (NCI) [10].

The purposes of this paper are as follows: (1) to present background information about online mailing lists and electronic support groups (ESGs), (2) to describe the rationale and methodology for the Health eCommunities (HeC) study, and (3) to present preliminary baseline data on new subscribers to cancer-related mailing lists who chose to respond to an online survey. In particular, we describe subscribers' use of mailing lists, their reasons for using them, and their reactions to participating shortly after joining.

### Use of the Internet for Support

The rise in Internet use has led to an increased number of people who seek support and information online. Some sources estimate that as many as 90 million Americans have participated in online support groups and that 1 in 4 people seeking disease information join online discussion groups [11]. Reportedly, 23 million Americans are very active in online communities [12]. A recent count found over 25000 health-related online self-help groups at one portal alone [13]. While the estimates vary greatly, whatever the correct number, millions of people in the United States turn to online support groups to deal with their health concerns.

Electronic support groups (ESGs), which include mailing lists, are much like self-help groups in that they are composed of members who share a common condition, situation, heritage, symptom, or experience [14]. They are self-governing, usually, with clear rules about acceptable behavior. ESGs range from highly structured therapeutic groups to moderated and unmoderated chat rooms and mailing lists. ESGs and mailing lists share the common goal of helping people learn about and cope with a variety of risk factors, diseases, and conditions. Some ESGs are closed groups with substantial professional moderation, such as those reported by Winzelberg et al [15] and Lieberman et al [14]. However, most of these moderated groups have been implemented as part of research projects. It is not clear whether such structures are viable as ongoing services that can be sustained over time.

Currently, most ESGs appear to be unmoderated and are more like self-help or mutual help groups than face-to-face support groups [16]. The mailing lists we are studying are characterized by wide reach and minimal intervention by most listowners who manage them. These lists are not moderated by health professionals although many of the listowners are extremely knowledgeable about health and cancer, and they intervene online and offline to correct misconceptions, enforce group norms, and provide information.

### Potential Positive Effects of Participating in ESGs

Patterson et al [17] identified three types of beneficial health outcomes for computer health care services: (1) education of people, (2) provision of social support or assistance in obtaining social support, and (3) change in health behaviors. Online support groups, including mailing lists, may provide both instrumental and social support [17-20].

Cancer patients use mailing lists and other Internet resources for many reasons. These include seeking and obtaining information and support, seeking second opinions, and getting information needed to interpret information from health providers [21]. In the process, the experience also may improve patients' self-esteem by putting them on a more comfortable basis with their health professionals. For patients with rare cancers, online groups may be the only way to get sufficient numbers of people together to form support groups [22]. By sharing practical advice with one another, users may gain the wisdom that experience brings [23].

ESGs may offer privacy and convenience, and people who do not feel well can participate from home. Moreover, people can communicate on the basis of shared experiences and concerns, not shared social characteristics (such as age, race, or gender) [14]. Internet-mediated support may be especially important for people who are geographically isolated and those with rarer cancers [24] and may be particularly valuable for minorities and people in rural areas because of documented disparities in their access to health care and health information [25]. Neuhauser and Kreps [26] cited other advantages of ESGs: the potential to be more interactive, participatory, and persuasive and to provide customized and contextualized information. Mailing list participation may have more reach and therefore greater population impact than in-person groups [27]. However, while there are a growing number of reports about patients' experiences with health-related mailing lists, to date, we are aware of no published outcome studies in this area.

Participation in ESGs may help patients be more involved in their care, find information, obtain support, and formulate questions to ask health providers [28]. Like other self-help groups, ESGs provide experiential knowledge and peer support [29]. Documented benefits of Internet applications range from decreases in pain and inappropriate health care use to improved quality of life [30]. Decreased anxiety and/or depression have resulted from both online therapy [31,32] and participation in ESGs for women with breast cancer [33]. Lieberman [14] also found positive increases in two subscales of the Posttraumatic Growth Inventory. Lorig et al [34] randomized arthritis patients to treatment consisting of a closed, moderated email discussion group plus book plus videotape or a control group. The treatment group had significant improvements in pain, disability, role function, and health distress and made fewer physician visits than the control group.

Access to health information through ESGs may serve an especially important communication function (eg, enhancing confidence in asking questions of one's physicians) [35]. Women with breast cancer who participated in the Computer Health Enhancement Support System (CHESS) were more competent seeking information, more comfortable participating in their care, and had greater confidence in their doctors. At five months follow-up, the group reported greater social support and information competence compared to nonusers of CHESS [36,37].

### Potential Limitations of Participating in Mailing Lists and ESGs

Online support groups have many of the same potential disadvantages as other forms of Internet communication, such as email. For example, there may be more hostile messages or “flaming” than would occur outside the Internet [22], and statements may be misinterpreted, causing discomfort and anxiety [22]. Offers of instrumental help are infrequent, and if people develop friendships, those relationships usually exist outside the mailing list where perhaps more instrumental social support can be provided [38]. Long-term relationships between individuals may be unusual [18]. There is still debate about whether computer use and, by extension, mailing lists/ESGs, promote social isolation, for example, by providing a more accessible but less sufficient substitute for meaningful social support [39]. Not surprisingly, mailing list postings include both information and misinformation. It is not known how these factors affect participants. In addition, some advice may encourage some people to adopt unconventional therapies [40]. As Lamberg [41] noted, finding the right ESG may take some work, and quality may vary even more than in community support groups. In the current milieu, selected messages may be blocked to protect against spam, potentially isolating some users.

The Science Panel on Interactive Communication and Health [42], while generally positive about the Internet, noted that there are some potential risks of using the Internet to obtain health information, including that patients could turn to inappropriate complementary and alternative treatments or that they could lose faith in their physicians. To date, there is little evidence of such effects [43]. Evaluations of Internet-based services should include both potential benefits and limitations. It is essential to understand both the strengths and limitations of mailing list participation.

### The State of the Evidence

Overall, Lieberman and Russo [14], based on a qualitative review of the literature, concluded that the impact of ESGs appears positive. Moreover, this seems to be a consequence of the qualities they share with in-person support groups (eg, people communicate with high levels of support, acceptance, positive feelings, normalization, and the perception of finding others like themselves and receiving meaningful information and support). Yet, as Eysenbach et al [13] concluded recently, based on a systematic review of all longitudinal studies, including cohort studies, before-after designs, and randomized trials, “No robust evidence exists for consumer-led peer to peer communities, partly because most peer to peer communities have been evaluated only in conjunction with more complex interventions or involvement with health professionals. Given the abundance of un-moderated peer to peer groups on the Internet, research is required to evaluate under which conditions and for whom electronic support groups are effective and how effectiveness in delivering social support electronically can be maximized” [13]. Today, therefore, the evidence is scant [13,23].

## Methods

### Health eCommunites Study Overview

**Figure 1:**
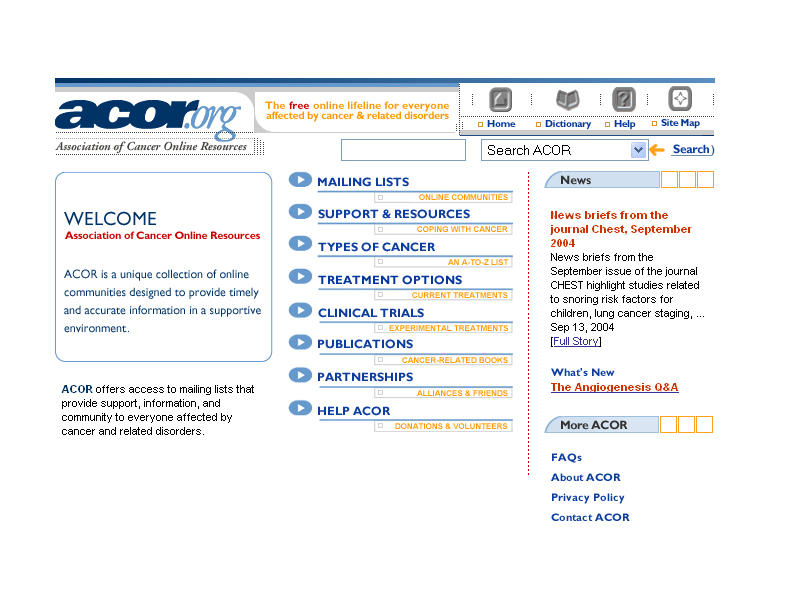
ACOR home page

Health eCommunities (HeC), funded through the Robert Wood Johnson Foundation's Health eTechnologies Program, is assessing the impact on cancer survivors and their caregivers of participating in mailing lists sponsored by the ACOR (Figure 1). HeC is based upon a partnership of ACOR and the School of Public Health at The University of North Carolina at Chapel Hill to assess the impact of participation in cancer-related mailing lists managed by ACOR. As part of the larger HeC study to understand the role that online cancer-related mailing lists play in the lives of people living and coping with cancer, we conducted an online survey of new cancer-related mailing list participants to address several research questions, including the following:

1. Why do new mailing list subscribers join cancer-related mailing lists?
2. What are new subscribers' expectations regarding mailing lists?
3. How frequently do new mailing list subscribers use the mailing lists?
4. How do they assess the lists shortly after joining, in terms of the similarity of their experiences to those of others and on several aspects of information seeking and social support?

The preliminary baseline data reported here were collected as part of a larger impact evaluation. Understanding why people join these mailing lists and how they use them will enhance what is known about online health support.

### Theoretical Approach

Our approach to understanding cancer survivors' use of cancer-related mailing lists is informed by theories on stress and coping [44-46] that emphasize primary appraisals of susceptibility to threats and perceptions of the severity of threats. Coping is the process of managing internal and external demands that are appraised as exceeding individual resources [47]. Lazarus and Folkman [48] identified two broad categories of coping strategies—problem focused and emotion focused. Either can lead to positive or negative outcomes. In problem-focused coping, also referred to as problem management, a person takes constructive action to deal with threats. This might include joining a cancer-related mailing list. In emotion-focused coping, also referred to as emotional regulation, a person acts to control emotional responses to a threat. This may include seeking social support, venting feelings, or practicing avoidance and denial.

Heightened perceptions of risk can cause distress, disengagement, and avoidance behaviors [44,45] but may also motivate problem management coping in the form of seeking information and social support. Current models of the processes by which people confront stress propose that a number of variables, such as personality characteristics, external resources, and social support, can influence coping, thereby mediating the effect of coping on psychological outcomes [49]. In particular, social support can facilitate an individual's positive efforts to cope, and it has the potential to bolster both positive problem-focused and emotion-focused coping. Further, the absence of a social support network has been tied to a more rapid course of illness (although these data should be received as preliminary) and to more depression [50]. Thus, seeking social support through mailing list participation may be a useful strategy that ultimately can improve a person's quality of life and health behaviors. Whether those factors can improve health outcomes remains to be seen.

### Survey Methods

We are using a longitudinal cohort design to assess the impact of ACOR mailing lists on selected outcomes for new subscribers; however, we only report cross-sectional baseline data in this preliminary paper. For the pilot study, new members were recruited to participate about a week after they subscribed to one of 10 ACOR mailing lists. (However, some subscribers may have waited several days to respond or may never have responded.) Invitations to participate were sent to new ACOR members via email. Willing participants could either follow hyperlinks to Web-based surveys or request that they be interviewed via telephone. As less than 1% of respondents requested telephone interviews, we will not discuss telephone interviews further in this paper. Up to three post-notification contacts were made to each non-respondent by email to increase responses [51,52]. All survey instruments and materials were reviewed by The University of North Carolina Institutional Review Board.

### Study Variables

For analyses reported here, we focused on information seeking and the processes by which new subscribers used the mailing lists. We also examined how new subscribers responded to the surveys through analysis of time-stamp data (described below). Relevant survey items are summarized as follows:

1. **Sociodemographic and medical variables.** We collected data on variables such as age, race, ethnicity, education, marital status, health insurance, and type of cancer. Because of sample size limitations, we did not examine differences among respondents according to these categories.
2. **Information seeking.** Information seeking items were drawn from the National Cancer Institute's Health Information National Trends Study (HINTS) [53].
3. **Mailing list use processes.** Adapting questions used by King [54], we collected information on the number of times subscribers read mailing list messages, the average number of minutes they spent reading/posting messages, how often they posted messages, and how often they contacted mailing list members outside of mailing list postings.

## Results

### Time-Stamp Data and Assessment of Nonresponse

Throughout the pilot phase, we collected data on survey usage patterns through time stamps. The online survey took a “stamp” of the time when respondents continued to another page of the survey, saved their progress, and completed the survey. See Table 1 for an example of time stamps for three respondents. These data were invaluable in revising surveys. During a pilot test, we cut over 1.5 minutes from the average survey completion time by adjusting pages that were particularly time consuming and demanding. We also reviewed where break-offs occurred and rearranged questions to maintain respondents' interest. When we saw that break-offs were clustered around a particular group of questions, we changed the order and broke those questions up into separate pages. Subsequently, break-offs became random and fewer, suggesting that time-stamp data permitted us to gain valuable insights into the way users were responding to surveys. The average time to complete the survey was 21.5 minutes. Data from incomplete surveys were excluded from the data reported here.

**Table 1:** Time-stamp data for three respondents*

**Page Number of Survey**	**Time of Page Submission**	**Page Time**	**Cumulative Time**	**Status**
1	6/17/04 5:42	New Record	0:00:00	
2	6/17/04 5:43	0:00:51	0:00:51	
3	6/17/04 5:44	0:00:21	0:01:12	
4	6/17/04 5:44	0:00:08	0:01:20	
5	6/17/04 5:44	0:00:23	0:01:43	
6	6/17/04 5:45	0:00:52	0:02:35	
7	6/17/04 5:46	0:00:54	0:03:29	
8	6/17/04 5:46	0:00:32	0:04:01	
9	6/17/04 5:47	0:00:21	0:04:22	
10	6/17/04 5:47	0:00:16	0:04:38	
11	6/17/04 5:47	0:00:27	0:05:05	
12	6/17/04 5:48	0:00:28	0:05:33	
13	6/17/04 5:49	0:00:46	0:06:19	
16	6/17/04 5:50	0:01:32	0:07:51	
17	6/17/04 5:51	0:00:47	0:08:38	
18	6/17/04 5:52	0:00:30	0:09:08	
19	6/17/04 5:53	0:01:15	0:10:23	
20	6/17/04 5:54	0:01:05	0:11:28	
21	6/17/04 5:54	0:00:16	0:11:44	
22	6/17/04 5:55	0:00:31	0:12:15	
23	6/17/04 5:55	0:00:09	0:12:24	Completed
				
1	6/17/04 7:43	New Record	0:00:00	Break-off at page 1
				
1	6/17/04 8:02	New Record	0:00:00	
2	6/17/04 8:03	0:01:11	0:01:11	
3	6/17/04 8:04	0:00:18	0:01:29	
4	6/17/04 8:04	0:00:12	0:01:41	
5	6/17/04 8:05	0:01:10	0:02:51	Break-off at page 5

^*^ One respondent completed the survey in 12:24 minutes, and two respondents broke off (one at less than 1 minute and one at 2:51 minutes).

### Response Rates and Challenges of Gaining Adequate Participation

**Figure 2:**
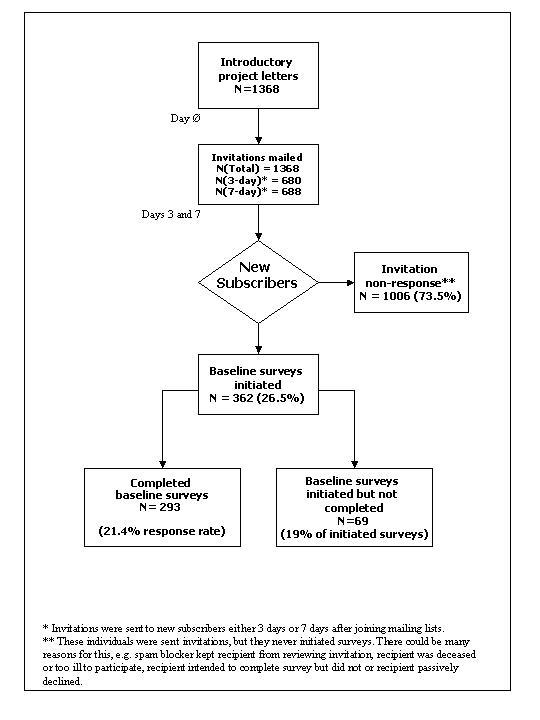
Project flow

Cumulative email invitations were sent to 1368 new mailing list subscribers; 293 Web surveys were completed (21.4% response rate). Figure 2 shows the flow from initial emails to survey distribution. Ideally, the response rate should be corrected for undeliverable email addresses and ineligibles. This is extremely challenging, much more so than for traditional survey methodologies, such as mailed surveys in which undeliverable letters can be obtained through the post office. It also is more difficult than for telephone surveys, in which ineligible numbers can be identified. When email invitations are sent, many will not be delivered for a variety of reasons, such as powerful spam blockers, changed email addresses, people who no longer use the mailing list but have not officially unsubscribed, and people who died or are now too ill to participate. Because people often turn to the lists when they are initially diagnosed or are suffering recurrences, subscribers may be preoccupied with doctors' visits and may have little discretionary time for other activities. Unfortunately, we cannot identify these sources of nonresponse.

### Survey Respondents

As Table 2 shows, most new mailing list subscribers were aged 50 or older (72%) and had insurance coverage (97%). Subscribers were nearly evenly divided between men and women. Most were married (80%), had no young children living in the household (79%), were white (98%), and had at least some college education (86%). The majority described their health as good or very good despite having had a cancer diagnosis. Most respondents were diagnosed with cancer in their 50s and reported being in treatment.

**Table 2:** Sociodemographic characteristics of respondents (N = 293)

**Characteristic**	Respondents^*^No. (%)
**Health Status**	
Excellent	40 (14)
Very good	90 (31)
Good	89 (30)
Fair	50 (17)
Poor	23 (8)
**Age**	
< 40	21 (7)
40s	60 (21)
50s	105 (37)
60s	71 (25)
≥ 70	27 (10)
**Gender**	
Male	149 (51)
Female	144 (49)
**Employment Status**	
Employed for wages	103 (35)
Self-employed	30 (10)
Out of work/unable to work	57 (20)
Homemaker/student/retired	101 (35)
**Marital Status**	
Married or living as married	233 (80)
Divorced/separated/widowed/never married	58 (20)
**Race**	
American Indian or Alaska native	2 (1)
Asian	3 (1)
Black or African American	1 (0)
Native Hawaiian or other Pacific Islander	0 (0)
White	286 (98)
**Highest Grade Completed**	
High school (grade 12), GED, or less	42 (14)
College, 1 year to 3 years (some college or technical school)	90 (31)
College, 4 years or more (college graduate)	161 (55)
**Have Medical Coverage**	
Yes	283 (97)
No	9 (3)

^*^ Percentages may not add to 100% due to rounding.

### Use of the Internet and Mailing Lists

We asked why respondents used the Internet over the past 30 days (Table 3). New members indicated that they often used the Internet to find out more about cancer (61%), to find information on general health issues (26%), and to communicate with others with the same condition (21%). In the last 30 days, 17% often used the Internet to find information on prescription drugs, and 12% often used the Internet to find information on health-related products such as herbal remedies and vitamins. Only 7% said they often used the Internet to communicate with doctors or other health professionals.

**Table 3:** Purpose of Internet use over past 30-day period (N = 293)

	Respondents^*^ No. (%)			
**Purpose**	**Not At All**	**Rarely**	**Sometimes**	**Often**
Find out more about cancer	11 (4)	13 (4)	91 (31)	176 (61)
Find information on general health issues	33 (11)	60 (21)	123 (42)	76 (26)
Communicate with other people who have the same condition	68 (24)	49 (17)	111 (38)	61 (21)
Find information on prescription drugs	75 (26)	69 (24)	97 (33)	49 (17)
Find information on health-related products such as herbal remedies and vitamins	105 (36)	82 (28)	70 (24)	35 (12)
Communicate with doctors or other health professionals (including email)	162 (56)	64 (22)	45 (15)	20 (7)

^*^ Rows may not add to 100% due to rounding.

New mailing list members said they used ACOR mailing lists for a variety of purposes (Table 4), including information and support. For example, respondents strongly agreed that they were participating in the mailing lists to find out about the latest cancer treatments (64%), to get information about how to deal with cancer (62%), to find out how to deal with side effects (53%), to get information about treatment options (53%), to see how other patients with the same cancer were doing (48%), and for support (42%). Even among these new subscribers, 37% strongly agreed they were on the mailing lists to help others. Less important reasons, but still strongly endorsed by one-third or more of respondents, were to get ideas about how to talk with doctors, to get help with decision making, and to reduce uncertainty.

**Table 4:** Reasons for using mailing lists (N = 293)

	Respondents^*^ No. (%)			
**Reason**	**Strongly Disagree**	**Disagree**	**Agree**	**Strongly Agree**
Find out about the latest treatments for cancer	6 (2)	9 (3)	89 (31)	184 (64)
Get information about how to deal with cancer	1 (0)	6 (2)	105 (36)	179 (62)
Find out how to deal with the side effects of cancer treatments	1 (0)	17 (6)	118 (40)	156 (53)
Get information about treatment options	6 (2)	11 (4)	120 (41)	153 (53)
See how other patients with my cancer are doing	3 (1)	12 (4)	137 (47)	138 (48)
Get support from other people with my cancer	6 (2)	30 (10)	133 (46)	121 (42)
Help me make decisions about what is the best cancer treatment for me	12 (4)	26 (9)	134 (46)	117 (41)
Help reduce my uncertainty about which treatments are best for me	12 (4)	32 (11)	131 (45)	114 (40)
Help others	3 (1)	26 (9)	153 (53)	109 (37)
Get ideas about how to talk with my doctor about my illness	11 (4)	41 (14)	135 (47)	103 (35)
Feel less lonely	28 (10)	73 (25)	100 (35)	87 (30)

^*^ Rows may not add to 100% due to rounding.

We asked these new subscribers to specific ACOR mailing lists how they used the mailing lists in the past seven days since they had subscribed. This information was intended to serve as the baseline for subsequent comparison: 78% said they checked their email for messages four or more times in the last seven days; 41% of new subscribers reported spending 1 to 3 hours per day reading and responding to mailing list messages; and 30% said they exchanged private emails with 1 to 3 subscribers (Table 5). Only small proportions of respondents reported private emails or phone calls with group members.

We also assessed reactions to group processes that took place on the mailing lists. Of new ACOR subscribers, 9% strongly agreed (62% agreed) that others on the list had similar experiences, 31% strongly agreed that they could express their opinions on the mailing lists, 27% strongly agreed that they could relate to other members' cancer experiences and that others on the mailing list gave them good ideas about how to cope with cancer, and 12% strongly agreed that they could disagree with other members' statements (Table 6). We asked how much help new members received from being on the mailing lists (Table 7): 39% indicated that other members gave them quite a bit/very much help, while only 7% said they gave quite a bit/very much help to other members. Fifty percent of new subscribers said listowners provided quite a bit/very much information that members needed, and 43% said that listowners helped with discussion quite a bit/very much.

**Table 5:** Mailing list use (N = 292)*

**Mailing List Use**	**Respondents** **No. (%)**				
	**0**	**1–3**	**4–6**	**7–9**	**10**
Number of times subscribers checked their mailing list email	41 (14)	23 (8)	60 (20)	87 (30)	81 (28)
Hours spent each day reading and responding to messages from mailing list	155 (54)	116 (41)	9 (3)	3 (1)	4 (1)
Number of different mailing list members with whom subscribers exchanged private emails	155 (54)	87 (30)	33 (12)	5 (2)	6 (2)
Number of times subscribers exchanged private emails with other mailing list members	177 (62)	65 (23)	27 (9)	7 (2)	12 (4)
Number of mailing list members subscribers called on the phone	269 (93)	18 (6)	1 (1)	0 (0)	0 (0)

^*^ Number may vary slightly due to skip patterns.

**Table 6:** Evaluation of mailing list experiences (N = 252)*

	**Respondents** **No. (%)**			
**Evaluation of Experiences**	**Strongly Disagree**	**Disagree**	**Agree**	**Strongly Agree**
**Cohesiveness**				
Overall, my experiences were similar to those of other members.	7 (3)	63 (26)	154 (62)	23 (9)
I could relate to other members' experiences about cancer.	3 (1)	19 (8)	160 (65)	66 (27)
**Normalization, Role Modeling**				
Other people on the mailing list gave me good ideas about how to cope with cancer.	3 (1)	33 (13)	147 (59)	66 (27)
**Conflict Management**				
I felt it was OK to express my opinions in the group.	2 (1)	18 (7)	149 (61)	77 (31)
I felt it was OK to disagree with other members' statements.	4 (2)	48 (20)	160 (66)	30 (12)

^*^ Number may vary slightly due to skip patterns.

**Table 7:** Help provided by the mailing lists (N = 276)*

	**Respondents** **No. (%)**				
**Help**	**None**	**ALittle Bit**	**Some**	**Quite a Bit**	**Very Much**
How much did the listowner (or listowners) give information that group members need?	18 (7)	27 (11)	80 (32)	80 (32)	46 (18)
How much did the listowner (or listowners) help the discussion?	31 (13)	27 (11)	83 (33)	71 (29)	36 (14)
How much help did other mailing list members give you?	27 (11)	32 (12)	97 (38)	67 (26)	33 (13)
How much did the listowner (or listowners) help group members resolve conflicts?	65 (28)	23 (10)	74 (32)	50 (21)	21 (9)
How much help did you give to other mailing list members?	125 (49)	48 (19)	63 (25)	16 (6)	3 (1)

^*^ Number may vary slightly due to skip patterns.

## Discussion

The picture that emerges from Web surveys completed by cancer survivors who were new subscribers and received invitations to participate within about a week after subscribing to ACOR mailing lists is one of people who turned to the mailing lists for information and support, especially information. They particularly were looking for information about treatment, coping with side effects, and treatment options, with half or more of new subscribers providing these as reasons for joining mailing lists. Over 40% also cited support as one of the reasons they joined the lists. Although only 37% of respondents noted helping others as one of the reasons they joined the list, it is striking that even this proportion of people saw helping others as a reason for joining mailing lists. It is possible that helping others and receiving support become more important over time as the lists become more useful and central to subscribers when their tenure on it increases. Reading and responding to messages accounted for one to two hours a day, on average, for survey respondents. Follow-up survey data will help to put these numbers in context. We cannot predict whether the amount of time will increase, decrease, or stay the same. Also, we do not know the accuracy with which people report time spent online.

In view of their newness to the lists, it would be expected that many new subscribers would watch and wait, perhaps even *lurk,* before playing an active role. Moreover, we would expect some of their assessments and perceptions to change over time. Several days into their use of a mailing list, many subscribers would not have observed conflicts or even be aware of listowners' actions to facilitate improved group processes. In fact, many of these interventions occur behind the scenes. Since this is a longitudinal study, we will be able to assess changes in participation patterns one and four months later as participants become more comfortable and perhaps more active on the mailing lists. Our larger study will also assess whether there are changes over time in subscribers' assessments of mailing list processes. We expect that the amount of help subscribers perceive themselves having received and given will be a function of many factors, including their illness trajectory, type of cancer, other support and information available to them, and the lists to which they subscribe.

### Limitations

Although our full study will examine changes over time in subscribers' use of mailing lists and other outcomes of interest, here we focused only on the preliminary baseline survey, which would have the same strengths and weaknesses as similar cross-sectional surveys. For example, we can only report what subscribers said and cannot infer causal relationships.

It is unfortunate that so few minorities completed surveys. We do not know if that is a reflection of a small number of minorities using mailing lists, their lesser inclination to complete this survey, or both. Recent data indicate that 46% of African American adults are online versus 64% of white and 63% of Hispanic Americans [2]. Thus, blacks still are using the Internet at a lower rate than whites but at a higher rate than our data would suggest. Fogel et al [55] found lower use of Internet listserv and self-help groups by minority breast cancer patients. McTavish et al [56] analyzed differences between black and white women who used online support. Black women were more likely to be lurkers, spent less time online, and wrote more messages about breast cancer and fewer messages about everyday life than white women. Klemm et al [57] reported that, in 10 studies they reviewed, most users of online support were white.

Only 14% of the respondents in Schmidt and Andrykowski's study of Internet support for breast cancer were African American [58]. However, the authors showed that Internet use for breast cancer information was associated with greater social support, and minorities showed greater gains than whites as a result of exposure to the intervention. Gustafson et al [36] and Shaw et al [24] similarly demonstrated that minority women with breast cancer benefit from use of the Internet. If, as Lieberman and Russo [14] concluded, ESGs are similar to face-to-face self-help groups in their beneficial impact, it may be appropriate to develop more proactive strategies to encourage diverse cancer survivors to use mailing lists and other ESGs.

More research is needed to understand the many issues involved in asking patients to complete Web surveys, from assessing the physical, psychological, and cognitive demands of different question formats to examining ways to estimate true response rates. Online and other survey methods are substantially different. The lessons of one method cannot be transferred to others without more research [59].

Our conservatively calculated response rate of 21.4% is less than one would expect based on other survey formats. However, as Kraut et al [59] and others noted, online surveys yield lower response rates than other survey methods. A recent report from RAND showed wide variation in response to Web-based surveys [60]. For example, Fogel et al [55] reported a 9% response rate of cancer patients to their online survey. Our results appear to be within the range of what has been reported for other online surveys of cancer patients. However, it is striking how few Internet-based survey reports contain response rates. It is likely that a substantial component of non-response may occur because of constraints inherent in online research, such as powerful spam blockers and changing email addresses.

### Unique Web-Based Tools

Finally, although there is much that is challenging about Internet research, especially when conducted among cancer survivors, the Internet also offers tools not available in other modes. For example, we found time-stamp data extremely valuable in overcoming break-offs by permitting us to pinpoint areas in the survey that respondents had trouble answering. Moreover, the potential to include people from around the world is an attractive aspect of Web surveys.

While our data do not yet answer Eysenbach's questions about the impact of online communities [13], the data do begin to paint a more complete picture of why cancer patients turn to the Internet, how they use mailing lists, and how they rate the processes of using the lists. As comments from subscribers show, this is a powerful world that is compelling and, potentially, not only supportive and informative but perhaps, sometimes, lifesaving as well. Creative research strategies will be needed to assess the many important questions related to use of online support by cancer patients and others. Among the intriguing questions are whether and how online support differs from in-person support and whether some people are more likely to benefit from one modality over another.

## Abbreviations

ACOR: Association of Cancer Online Resources
CHESS: Computer Health Enhancement Support System
ESGs: electronic support groups
HeC: Health eCommunities
RWJF: Robert Wood Johnson Foundation
